# Self-referral and associated factors among patients attending adult outpatient departments in Debre tabor general hospital, North West Ethiopia

**DOI:** 10.1186/s12913-021-06642-7

**Published:** 2021-06-28

**Authors:** Tigist Misganaw Abere, Desta Debalkie Atnafu, Yaread Mulu

**Affiliations:** 1 College of Medicine and Health Science, Bahir Dar University, Bahir Dar, Ethiopia; 2 Department of Health System & Health Economics, Bahir Dar University College of Medicine and Health Science, School of Public Health, Bahir Dar, Ethiopia

**Keywords:** Self-referral, Referral system and Ethiopia

## Abstract

**Background:**

Self-referral leads to diminished quality of health care service; increase resource depletion and poorer patient outcomes. However, a significant number of patients referred themselves to the higher health care facilities without having referral sheets globally including Ethiopia. Even though the problem is much exacerbated in Ethiopia, there is limited evidence regarding self-referral patients in Ethiopia in particular in the study area.

**Objective:**

To assess the magnitude and associated factors of self-referral among patients at the adult outpatient department in Debre Tabor general hospital, North West Ethiopia.

**Method:**

Institution-based cross-sectional study was conducted from March 11–April 9, 2020 among 693 patients who attended adult outpatient departments. A systematic sampling technique was employed. Structured and pretested interviewer-administered questionnaire was used for data collection. Data were coded, cleaned and entered into Epi Info version 7.1 and exported to SPSS version 23 for further analysis. Binary logistic regression analysis was employed. In bivariable analysis *p*-value, less than 0.25 was used to select candidate variables for multivariable analysis. *P*-values less than 0.05 and 95% confidence intervals were used to select significant variables on the outcome of interest.

**Result:**

The proportion of self-referral was 443(63.9%) with 95% CI (60.5; 67.5). Formally educated, (AOR = 1.83; (95% CI: 1.12, 3.01)), enrolled to Community Based Health Insurance (AOR = 1.57; (95% CI: 1.03, 2.39)), poor knowledge about referral system (AOR = 2.07; 95% CI: (1.28, 3.39)), not and partially available medication in the nearby Primary Health Care facilities (AOR = 2.12; (95% CI: 1.82, 6.15)) & (AOR = 3.24; (95% CI: 1.75, 5.97)) respectively and history of visiting general hospital (AOR = 1.52; (95%CI: 1.03, 2.25)) were factors statistically associated with self-referral.

**Conclusion and recommendation:**

The proportion of self-referral was low compared to the Ethiopian health sector transformation plan 2015/16–20. Socio-demographic and institutional factors were associated with self-referral. Therefore, regional health bureau better to work to fulfill the availability of medications in the primary health care facilities. In addition, Community Based Health Insurance (CBHI) agency should work to implement the law of out-of-pocket expenditure which states to pay 50% for self-referred patients who claim utilization of healthcare.

## Plain English summary

An effective referral linkage is an integral component of a successful health care system for quality health service. Many developing countries have policies regarding referral system while transforming referral policies into practice between primary health care (PHC) facilities and higher-level facilities is challenging. This study was trying to answer the magnitude and factor associated with self-referral through structured interview questionnaires.

The participants were asked about their socio-demographic characteristics, institutional related characteristics.

There were 690 participants in this study. This study showed that the magnitude of self-referral was 63.9%. Educational status, knowledge about referral system, availability of medication in the nearby PHC facilities, enrollment to CBHI and history of visiting general hospital were factors significantly associated with self-referral.

In conclusion, the proportion of self-referral was low compared to the Ethiopian health sector transformation plan 2015/16–20. Educational status, knowledge about referral system, availability of medication in the nearby PHC facilities, enrollment to CBHI and history of visiting general hospital were associated with self-referral. Policy action will be required to further improve ANC service utilization.

## Introduction

A referral system entails the interrelationship and coordination of patient care services from one health care facility to another. a referral is a process by which a health care worker transfers the responsibility of care temporarily or permanently to another health professional in response to their inability or limitation to provide the necessary care [1]. While self-referral is a situation when patients refer themselves to higher-level health care facilities other than the primary care facilities without having referral sheets [2].

An effective referral linkage is an integral component of a successful health care system for quality health service. Many developing countries have policies regarding to the referral system while transforming referral policies into practice between primary health care (PHC) facilities and higher-level facilities is challenging. To strengthen referral system all levels of the health care delivery system need to be functioning appropriately. In developing countries, higher-level health care facilities were overcrowded with patients who could be treated in lower level facilities which is a common feature of a poorly functioning referral system [3–6].

Self-referral causes depletion of resources such as patients waiting long hours and wasting of highly trained medical workers’ time for minor cases. As a result, patients frequently referred to another hospital and die on the way. Moreover, due to the large patient load, human and physical resources are stretched to capacity, which results in hospitals compromising the care that they provide to patients [7].

The Ethiopian government has made remarkable progress to improve access to PHC units for all. According to the 2015 World health organization report, 1.6 billion dollars was financed for health care, of the total health expenditures, 14.69% goes to finance PHC. However, many patients attend a higher level of care for their initial visit that can be managed at a lower level without having a referral sheet [8–10]. As result, secondary level hospitals were congested and overburdened [2].

Studies conducted in Africa showed that the magnitude of self-referral was 27.7, 30.8 33.9, 60, 87 and 96.1% in Nairobi Kenya, Mozambique, Ghana, Nigeria, Sudan and Kirinyaga district Kenya respectively [11–16]. In addition, the studies done in South Africa showed that the magnitude of self-referral was 35, 36, 86.9 and 88.2% in Kwazulu Natal, EThekwini District, Tubatse local Municipality and Western Cape province of respectively [17–20]. Similarly, studies done in Ethiopia the magnitude of self-referral in general and referral hospitals were 82 and 84.4% respectively [2, 21]. Additionally study conducted in Hosanna town, Hadiya Zone shows that 67% mothers bypassed their catchment public health centers [22].

Studies conducted in Sub Saharan Africa showed that respondents whose age is 40 years and below, sex of the respondents, income, educational level, distance, waiting time, availability of diagnostic test and medication, knowledge about the referral system and access to transportation were the factors that influence patient self-referral [11, 14, 15].

Similarly, studies conducted in Ethiopia showed that access to transportation, availability of laboratory service, availability of prescribed drugs and obtaining information about the referral system from health care worker at the nearby PHC facilities affected patient self-referral However, the relationship between self-referral and community based health insurance (CBHI) were not assessed [2, 6, 21, 23].

There are limited findings with regards to self-referral in our country and the study area. Therefore, the aim of this study was to assess the magnitude and factors associated with self-referral among patients at adult outpatient departments in Debre Tabor General Hospital.

## Methods

### Study area

The study was conducted in Debre Tabor comprehensive specialized hospital previously called Debre Tabor general hospital before September 2020. It is located in south Gondar zone which is 670 Km far from Addis Ababa the capital city of Ethiopia. According to the Federal democratic republic of Ethiopia central statistics agency population projection of 2014–2017 reports, the total population of the South Gondar Zone was 2,484,929 of whom 1,257,323 are men and 1,227,606 women [24]. There are 405-health posts, 96-health centers, eight primary hospitals and one comprehensive specialized hospital. The total population served by this hospital is 2.3 million peoples. The hospital catchment area includes four town administrations and 14 districts [25]. An institution-based cross-sectional study design was employed from March 11–April 9, 2020. All patients who attended the adult outpatient department in Debre Tabor General Hospital were our source population and selected patients who attended the adult outpatient department in Debre Tabor General Hospital were our study populations. Patients whose age 15 and above who visited adult outpatient departments during the study period were included while those patients who are critically ill/ unable to respond were excluded.

### Sample size and sampling procedure

The sample size was determined by using both single population proportion formula and factor analysis while we found better sample size on objective one; based on the following assumptions, with Proportion of self-referral patients =82% [2], margin of error (3%), 95% CI. After adding 10% nonresponse rate, the final sample size was 693.

To recruit each participant’s systematic sampling technique was employed by considering an average monthly patient flow of the hospital 6302 patients per month. The first participant was identified by lottery methods. Finally, every patient with 9th interval was included in the study until the final sample sizes were obtained.

### Study variables

#### Dependent variable

Self-referral (Yes/No).

##### Independent variables

Socio-demographic factors (Sex of the respondent, age of the respondent, wealth index, educational status, occupation, marital status and residence), Individual factors (Knowledge about the referral system, perceived severity of illness and perceived treatment at the general hospital is better) and Institutional factors (Distance, waiting time at PHC facilities, availability of diagnostic and medication at the nearby PHC facilities, access to transportation and obtaining information about referral system from health care workers).

#### Operational definitions

##### Self-referral

self-referral is a situation when patients refer themselves to higher-level health care facilities (General Hospital) first before they visit primary health care facilities.

##### Knowledge about the referral system

eight questions were used to measure the knowledge of patients with regard to the referral system. If the patients answer more than 75% of the knowledge question he/she was considered as having good knowledge, if the patients answer 45 to 75% he/she was considered as having fair knowledge and if they answer less than 45% they were considered as having poor knowledge [26].

##### Wealth index

The socioeconomic status of each household was constructed using principal component analysis (PCA) of household assets followed by stratification of the households into wealth quintiles. The analysis was done by aggregating the ownership of durable assets; access to utilities and infrastructure; and housing characteristics; ownership of land and ownership of livestock variables into a single proxy variable of household wealth. All asset variables were coded into binary variables. Asset variables with zero standard deviations were excluded from the PCA as they did not contribute to the analysis. The first component of the PCA was used to construct the wealth quintiles. Based on the PCA weights for each asset variable, an aggregated score was calculated for each of the surveyed households, which was grouped into quintiles with quintile 1 (Q1) representing the poor 33% of households in the sample and quintile 3 (Q3) representing 33% of the better-off (rich). The study subjects were thereafter grouped into quintiles based on their household wealth [27–29].

#### Data collection tools and procedures

Structured and pretested interviewer-administered questionnaires were developed by reviewing different literatures [12, 14, 15, 21, 30, 31]. First, the questionnaires were prepared in English then translated into the local language Amharic and translated back to English to check the consistency. The questionnaire consists of socio-demographic, individual and institutional factors. Data were collected via interview before obtaining service in the out-patient waiting area and training was provided for both data collectors and supervisors. Three-degree holder Nurses was recruited for data collection and one master holder were assigned for supervising data collection along with the principal investigator.

#### Data quality control and assurance

The Amharic version of the questionnaires was used to collect the data. A aretest was conducted among 70(10%) patients at Enjibara General Hospital before the study period to check the consistency of the questionnaire. Two days training was provided for data collectors and supervisors on the objective, the purpose, how to keep confidentiality, how to approach patients and how to take consent. The filled questionnaires were checked every day by the supervisor and every week by the principal investigator for completeness and consistency.

#### Data analysis and management

Data were coded, entered and cleaned in to Epi-Info version 7.1 and exported to SPSS version 23 for further analysis. Descriptive statistics (frequency, percentage, SD and mean) were employed to summarize the variables. Binary logistic regression analysis was employed to see the relationship between dependent and independent variables. Bivariable analysis was used to select the candidate variable for multivariable analysis at *p* value less than 0.25. Variance inflation factors (VIF) were used to check Multi-collinearity. *P*-values and confidence intervals were used to select significance variables on multivariable analysis and those variables whose *p*-value less than 0.05 were considered as statistically significant. Hosmer and Lemeshow test was used to check the model fitness.

## Results

A total of 693 patients participated in this study with a response rate of 100%.

### Socio-demographic characteristics of the respondents

Two hundred ninety four (42.4%) respondents were in the age groups of 35 and above years with the mean age and standard deviation of 34.3 ± 11.9 years. The majority of respondents (52.4%) were living in urban. Two hundred sixty nine (38.8%) respondents were not attended formal education while 98 (14.1%) respondents attained degree and above. Two hundred thirty two (33.5%) respondents were from the rich family wealth index. Two hundred sixty seven (38.5%) respondents have good knowledge about referral system Three hundred sixty (51.9%) respondents were not enrolled in CBHI (Table 1).

**Table 1:** Socio-demographic characteristics of the participants in Debre Tabor general hospital, 2020 (*n* = 693)

Variables	Frequency (%)
**Age of the respondents**	
15 to 24	156 (22.5)
25 to 34	243 (35.1)
35 & above	294 (42.4)
**Sex of the respondents**	
Female	350 (50.5)
Male	343 (49.5)
**Marital status**	
Single	206 (29.7)
Married	426 (61.5)
Widowed	38 (5.5)
Divorced	23 (3.3)
**Educational status**	
No formal education	269 (38.8)
Primary	90 (13.0)
Secondary	115 (16.6)
College diploma	121 (17.5)
Degree & above	98 (14.1)
**Occupation**	
Governmental employee	99 (14.3)
Merchant	124 (17.9)
Farmer	223 (32.2)
Student	120 (17.3)
Others	127 (18.3)
**Wealth Index**	
Poor	232(33.5)
Middle	229(33.0)
Rich	232(33.5)
**Knowledge**	
Poor	202(29.2)
Fair	224(32.3)
Good	267(38.5)
**Enrollment to CBHI**	
Yes	333(48.1)
No	360(51.9)

Others = Daily laborers, NGO, Brokers, Drivers, Unemployed, house wife, and Tailor

### Institutional factors

Five hundred two (72.4%) respondents replayed that the health center is the closest health facility to their place of residence. Four hundred fifty two (70.8%) respondents received information about the referral system from health care providers. Four hundred sixty seven (67.4%) respondents have access to transportation. Four hundred thirteen (59.5%) of the respondents have used a car as a means of transportation (Table 2).

**Table 2:** Institutional related characteristics of the respondents in Debre Tabor general hospital, 2020 (*n* = 693)

Variables	Frequency (%)
**Nearby health facility closest to home**	
Health center	502 (72.4)
Primary hospital	155 (22.4)
General hospital	36 (5.2)
**Visit nearby health facility for current health problem**	
Yes	551 (79.5)
No	142 (20.5)
**Availability of medication at PHC (*n*** **= 551)**	
All available	78 (14.2)
Some available	264 (47.9)
None available	209 (37.9)
**Availability of laboratory at PHC (*n*** **= 551)**	
All available	65 (11.8)
Some available	178 (32.3)
None available	308 (55.9)
**Waiting time at PHC (*n*** **= 551)**	
Too short	326(59.2)
Too Long	225(40.8)
**Distance from the hospital**	
Less than one hour	253(36.5)
One to two hour	67(9.7)
More than two hour	373(53.8)
**Access to transportation**	
Yes	467 (67.4)
No	226 (32.6)
**Mode of transportation**	
Car	413 (59.5)
Animal	105 (15.2)
On foot	175 (25.3)

### Magnitude of self-referral

The magnitude of self-referral in this study was 443 (63.9%) with 95% CI (60.5; 67.5). The main reasons for self-referral were expected to get better treatment at the general hospital 63.1%, not expected to get laboratory investigation 58.7 and 49.9% not expected to get medication at the nearby PHC facilities.

### Factors associated with patients self-referral

In bivariable analysis educational status, place of residence, wealth index, history of visiting the general hospital, enrollment to CBHI, knowledge about referral system, distance to the health facilities, accessibility of transport, availability of medication at the nearby PHC facilities, waiting times at PHC facilities were found be candidate variable for multivariable analysis at *p*-value less than 0.25. On multivariable analysis educational status, history of visiting general hospital, Enrollment to CBHI, knowledge about referral system and availability of medication at nearby PHC facilities were statistically significant at *p*-value less than 0.05.

The odds of self-referral among patients who attend formal education was 1.83 times (AOR 1.83; (95%; CI; 1.12, 3.01)) higher compared to those who did not have formally educated. The odds of self-referral among patients who were enrolled in CBHI was 1.57 times (AOR 1.57; (95%; CI; 1.03, 2.39)) higher as compared to those who were not enrolled to CBHI. The odds of self-referral among patients who have poor knowledge about the referral system was 2.07 times (AOR 2.07; 95% CI (1.28, 3.39)) higher compared to those patients who have good knowledge (Table 3).

**Table 3:** Factor associated with self-referral among patients who attend Debre Tabor general hospital OPDs, 2020

Variable	Self-referral		COR (95% CI)	AOR (95% CI)
	Yes	No		
**Educational status**				
No formally educated	131	138	1	1
Formally educated	312	112	2.94(2.13,4.05)	1.83(1.12, 3.01)*
**Residence**				
Urban	273	90	2.86(2.07, 3.94)	1.19(0.62, 2.31)
Rural	170	160	1	1
**Enrollment to CBHI**				
Yes	275	85	3.18(2.29,4.39)	1.57(1.03,2.39)*
No	168	165	1	1
**Knowledge about referral system**				
Poor	152	50	2.41(1.62, 3.59)	2.07(1.28, 3.39)*
Fair	142	82	1.37(0.95, 1.97)	1.24(0.93, 2.24)
Good	149	118	1	1
**Wealth Index**				
Poor	116	116	1	1
Middle	153	76	2.01(1.38, 2.93)	0.81(0.39, 1.66)
Rich	174	58	3.00(2.03, 4.45)	1.18(0.69, 2.02)
**Distance**				
< One hour	215	158	1	1
1 to 2 h	44	23	1.41(0.82, 2.42)	1.09(0.67, 1.76)
2 and more hour	184	69	1.96(1.39, 2.77)	1.43(0.73, 2.84)
**Availability of medication (*n*** **= 551)**				
All available	28	50	1	1
Some available	174	90	3.45(2.04,5.85)	3.24(1.75, 5.97)*
None available	123	86	2.55(1.49,4.38)	2.12(1.82, 6.15)*
**Visiting General hospital previously**				
Yes	251	110	1.66(1.22,2.27)	1.52(1.03, 2.25)*
No	192	140	1	1

The odds of self-referral among patients who replied that medication is not available at all and some medication is available at the nearby primary health care facilities were 2.12 (AOR 2.12; (95% CI; 1.82, 6.15)) and 3.24 (AOR 3.24: (95% CI; 1.75, 5.97) times greater compared to those who have got all the medication at nearby PHC facilities respectively. The odds of self-referral among patients who had a history of visiting General hospital was 1.52 times (AOR, 1.52; (95%CI; 1.03, 2.25) higher compared to those who did not visit (Table 3).

## Discussion

The magnitude of self-referral in the study area was 63.9% with 95% CI (60.5; 67.5). This finding was consistent with a study conducted in Hadya Zone Ethiopia which shows that 67% [22] of the patients were self-referral. However, the proportions of self-referral in this study was lower compared with studies conducted in western Ethiopia 84% [21] and 82% [2]. This difference might be attributed to the intervention that the Ethiopian HSTP focus to improve the accessibility and quality of PHC facilities since 2016 [8]. Moreover, the finding of this study was higher than a study conducted in South Africa which was 35% [18] of patients in the outpatient department were self-referred. This may be due to in south Africa different family health service specialists assigned at PHC service to provide comprehensive specialty care to the community [32] while in Ethiopia specialty care is only delivered at higher health care facilities which are beyond the PHC level [8]. The Ethiopian government may take this lesson and should diversify healthcare services provided at the primary healthcare level in order to avoid unnecessary referrals.

The finding of this study showed that patients who attend formal education were more likely to be self-referred to general hospitals compared to those who did not attend formal education. The finding of this study was consistent with study conducted in India [31]. This might be due to educated patients were perceived their illness to be unpredictable with worse outcomes [33] because of this they need more specialized care. In addition, education is one of the means to increase ones household income and they are more capable to spend money on the medical expenses [34]. And when people are educated they would know that better healthcare can be received in higher level health facilities.

This study identified patients who enrolled in CBHI were more likely to be self-referred compared to their counterparts. There is limited finding with the relationship between CBHI and self-referral. The reason for this might be patients who enrolled in CBHI cover low out-of-pocket payments for medical expenses hence they prefer high level and specialized health care service [35]. Thus, facilities should prohibit services for peoples with self-referrals who treated free of charges due CBHI claim right, Similarly CBHI agency should devise mechanisms of halting such unnecessary referrals.

The findings of this study showed that patients who have poor knowledge about the referral system were more likely to be self-referred compared to those patients who have good knowledge about the referral system. This finding was consistent with studies conducted in Nigeria [14] and Iran [36]. This might be due to knowledge is one of means to increases the understandings of the patients about the general service provision of the facilities and the chains of lower to higher health care facilities. Moreover, they are more likely to understand the existing referral system [37].

Patients who visit General hospital previously were more likely to be self-referred themselves compared to those who did not visit the service previously. This finding was consistent with a study conducted in Ghana [13]. The possible reason for this is they are more familiar with the provision of the services at the general hospital.

This study identified availability of medication at PHC facilities was associated with patient’s self-referral. The finding of this study was in line with studies conducted in Ethiopia [2, 21] and South Africa [20]. This might be due to patients were more prefer to use facilities with available resources. Availability of health care resources in the facilities more attract the health care service users [38]. This implies that fulfilling the availability of medications at the lower health care facilities was reducing patient’s self-referral.

### Limitation and strength

This study might be prone to recall bias due to some of the variables assesses the respondents’ previous experiences this may lead us the false results. To minimize these patients were asked the recent visiting experience of the facility.

## Conclusion

This study shows that the proportion of self-referral in Debre Tabor general hospital was lower compare to the Ethiopian health sector transformation plan 2015/16–20 which stated that all individuals passed through primary health care services. Educational status, knowledge about referral system, availability of medication in the nearby PHC facilities, enrollment in CBHI and history of visiting general hospital were factors significantly associated with self-referral. Community-Based Health Insurance (CBHI) agency should work to implement the law of out-of-pocket expenditure which states to pay 50% for self-referred patients who claim utilization of healthcare.

## Data Availability

The dataset/raw data used and/or analyzed during the current study available from the corresponding author on reasonable request.

## Abbreviations

AOR: Adjusted Odds Ratio
CBHI: Community Based Health Insurance
CI: Confidence Interval
COR: Crude Odds Ratio
FMOH: Federal Ministry of Health
HSTP: Health Sector Transformation Plan
OPD: Out Patient Department
PCA: Principal Component Analysis
PHC: Primary Health Care
